# Role of LncRNAs and CircRNAs in Bone Metabolism and Osteoporosis

**DOI:** 10.3389/fgene.2020.584118

**Published:** 2020-11-13

**Authors:** Suryaji Patil, Kai Dang, Xin Zhao, Yongguang Gao, Airong Qian

**Affiliations:** ^1^Lab for Bone Metabolism, Xi’an Key Laboratory of Special Medicine and Health Engineering, Key Lab for Space Biosciences and Biotechnology, Research Center for Special Medicine and Health Systems Engineering, NPU-UAB Joint Laboratory for Bone Metabolism, School of Life Sciences, Northwestern Polytechnical University, Xi’an, China; ^2^School of Pharmacy, Shaanxi Institute of International Trade & Commerce, Xi’an, China; ^3^Department of Chemistry, Tangshan Normal University, Tangshan, China

**Keywords:** bone metabolism, long ncRNAs (lncRNAs), circular RNA (circRNAs), osteoporosis, therapeutics

## Abstract

Bone is a mechanosensitive organ that provides strength and support. Many bone cells, various pathways, and signaling molecules coordinate bone metabolism and also determine the course of bone diseases, such as osteoporosis, osteonecrosis, osteopenia, etc. Osteoporosis is caused by increased bone resorption and reduced bone formation due to the changes in the level of different proteins and RNAs in osteoclast or/and osteoblasts. The available therapeutic interventions can significantly reduce bone resorption or enhance bone formation, but their prolonged use has deleterious side effects. Therefore, the use of non-coding RNAs as therapeutics has emerged as an interesting field of research. Despite advancements in the molecular field, not much is known about the role of long non-coding RNAs (lncRNAs) and circular RNAs (circRNAs) in bone homeostasis and osteoporosis. Therefore, in this article, we summarize the role of lncRNAs and circRNAs in different bone cells and osteoporosis so that it might help in the development of osteoporotic therapeutics.

## Introduction

**FIGURE 1:**
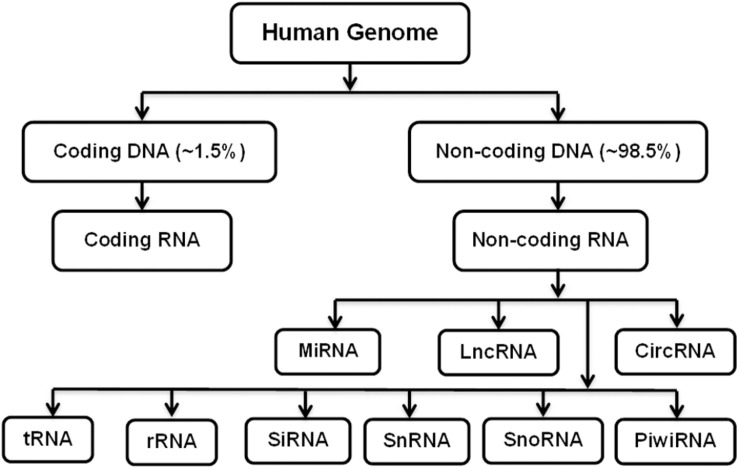
Coding and Non-coding RNAs.

Osteoporosis is characterized by reduced bone mass, weakened microarchitecture, and risk of fractures, affecting both women and men (Lane et al., 2000). More than 200 million people are estimated to be suffering from osteoporosis worldwide. In their lifetime, 1 in 3 women and 1 in 5 men worldwide become susceptible to osteoporotic fractures, making it one of the life-threatening diseases (Sözen et al., 2017). Different factors contribute to the pathophysiology of osteoporosis, such as defects in trabecular microarchitecture, inability to repair damage caused by normal activities, and increased rate of bone remodeling (Armas and Recker, 2012). Dual-energy X-ray absorptiometry (DEXA) and quantitative computed tomography (CT) scans are the common diagnostic techniques used to estimate bone mass and risk of fractures (Lane et al., 2000). Pharmaceutical treatments available for osteoporosis include calcium and vitamin D, calcitonin, estrogen replacement therapy (ERT), etidronate, fluoride, intermittent parathyroid hormone therapy (iPHT), and bisphosphonates alendronate (ALN) and risedronate (RIS) to reduce the bone resorption or increase bone formation (Prestwood et al., 1995; Eiken and Vestergaard, 2016). Despite these advantages, these pharmaceutical agents alone or in combination have limitations, such as low efficacy in reducing non-vertebral fracture and adverse effects of long-term application (Tabatabaei-Malazy et al., 2017). Therefore, the identification and development of new therapeutics based on gene therapy, which employs the use of various methods to deliver different nucleic acids, such as DNA or RNA, into the cells has gained significant attention. The nucleic acid used can be coding or non-coding. Non-coding RNAs (ncRNAs) are non-translated RNAs that regulate the expression of various genes and are associated with different biochemical pathways, DNA repair, and genome integrity as well as cellular function. They are commonly abnormally expressed or functionally flawed in diseases. The progress in the complexity and quality sequencing of transcriptomes, such as RNA-Seq, has allowed the profiling of transcriptomes, and with advancements in RNA-Seq, the identification of novel and diverse ncRNAs has become possible (St Laurent et al., 2015; Arrigoni et al., 2016). They comprise small interfering RNAs (siRNAs), microRNAs (miRNAs), PIWI-interacting RNAs (piRNAs), small nucleolar RNAs (snoRNAs), long non-coding RNAs (lncRNAs), circular RNAs (circRNAs), etc. (Wan et al., 2014; Sandhu et al., 2016; Figure 1). The dysregulation in these ncRNAs is found to have relevance to various diseases, including cancer, such as gastric cancer, and immunological, cardiovascular, developmental, and other diseases, such as bone diseases (Esteller, 2011; Huang et al., 2013; Zhang and Du, 2016; Ding et al., 2018; Chen et al., 2019d; Yu et al., 2020).

Except for miRNAs, the role of many other ncRNAs, such as lncRNAs or circRNAs, has not been exploited very much in bone metabolism and osteoporosis.

Despite their inability to code for any proteins, non-coding RNAs can influence the expression of other genes through several mechanisms; some of which are well known and well established, and others are not (Matsui and Corey, 2017). This influence makes them an ideal target for drug development with minimal side effects. Therefore, in this review, we summarize the role of ncRNAs, especially lncRNAs and circRNAs, in bone cells affecting bone metabolism and osteoporosis.

## The Role of lncRNAs

Long non-coding RNAs constitute a group of RNA transcripts that do not code for any protein and are characterized by the presence of more than 200 nucleotides. The expression of the target gene is regulated by lncRNAs through *cis*- or *trans*-regulation (Chi et al., 2019). Due to their ability to epigenetically control the expression of various genes in physiological as well as pathological processes makes them a critical factor in human diseases, such as osteoporosis (Wu et al., 2018b; Chi et al., 2019; Yu et al., 2019).

### LncRNAs in Bone Mesenchymal Stem Cell Regulation

Mesenchymal stem cells (MSCs) provide a vital source in regenerative medicine as well as cell therapies in advanced-age patients. Bone marrow MSCs (BMSCs) provide support for hematopoietic cells as well as play an important part in bone formation, and this distinctive ability makes them unique and possibly sensitive to age-related diseases (Baker et al., 2015). Therefore, it is important to study the molecular mechanism involved in BMSC regulation (Table 1).

**TABLE 1:** LncRNAs involved in BMSC regulation.

LncRNA	Relative expression	Role	Target	References
HOTAIR	Upregulation	Inhibits adipogenic differentiation of MSCs	DNA methylation	Kalwa et al., 2016
SEMA 3B-AS1	Upregulation	Inhibits proliferation and osteogenic differentiation of hMSCs	Proteins in actin cytoskeleton, FA	Zhang et al., 2019a
XIST	Upregulation	Inhibits osteoblast differentiation	Runx2, ALP	Chen et al., 2019c
AK016739	Downregulation	Promotes bone formation in osteoporosis mice	–	Yin et al., 2019
BDNF-AS	Upregulation	Inhibits osteogenic differentiation of BMMSCs	BDNF	Feng et al., 2018b
Bmncr	Upregulation	Stimulates bone formation *in vivo*	fibromodulin	Li et al., 2018a
Bmcob	Upregulation	Promotes osteoblastic differentiation of BMSC	SBP2	Sun et al., 2018
NEF	Upregulation	Promotes osteoporosis	IL-6	Ma et al., 2019

*“–” Unknown.*

In cancer cells, lncRNA HOX transcript antisense RNA (HOTAIR) is known to regulate gene expression by mediating the assembly of chromatin modifiers (Loewen et al., 2014). In MSC regulation, upregulated HOTAIR is shown to inhibit MSC differentiation into adipogenic cells by modulating RNA–DNA–DNA triple helix formation, and increasing cell passage could hypermethylate the HOTAIR binding sites at genomic regions, facilitating this triple helix formation (Kalwa et al., 2016). Thus, through epigenetic modifications, HOTAIR could regulate the MSC differentiation Antisense lncRNAs are also involved in regulating osteogenic differentiation of human mesenchymal stem cells (hMSC)s, such as semaphorin 3B-Antisense 1 (SEMA3B-AS1). The lentivirus-mediated upregulation of SEMA3B-AS1 is revealed to downregulate the expression of proteins involved in the actin cytoskeleton and focal adhesion (FA) as well as those that are involved in extracellular matrix–receptor interaction. This downregulation inhibited proliferation and osteogenic differentiation of hMSCs (Zhang et al., 2019a). Another antisense lncRNA involved in bone metabolism is lncRNA brain-derived neurotrophic factor antisense (BDNF-AS). During the osteogenic differentiation of BMSCs, the expression of lncRNA BDNF-AS was elevated. A gain-of-function experiment shows BDNF-AS prompted BMSCs to proliferate but did not promote osteogenic differentiation. Furthermore, it is suggested that the upregulation of BDNF-AS downregulated the levels of BDNF, osteopontin (OPN), and runt-related transcription factor 2 (Runx2), thus reducing osteogenic differentiation (Feng et al., 2018b).

The detection of initial BMSC differentiation into osteogenic cells is generally measured by the activity of alkaline phosphatase (ALP) *in vitro* and also serves as an important factor in the calcification of BMSCs (Chen et al., 2019c). It has been described that the high expression of lncRNA X-inactive specific transcript (XIST) had a negative effect on ALP and Runx2 expression and osteoblast differentiation in BMSCs (Chen et al., 2019c). The high expression of lncRNA AK016739 in BMSCs has also been described to negatively correlate with marker genes of osteogenic differentiation. This high expression of AK016739 inhibited osteoblast differentiation, whereas *in vivo* inhibition rescued the bone formation in ovariectomized (OVX) osteoporosis mice (Yin et al., 2019). According to Li et al., lncRNA, bone marrow associated ncRNA (BMNCR) could also regulate the fate of BMSCs through fibromodulin, an extracellular matrix protein, and activating the bone morphogenetic protein (BMP) pathway (Li et al., 2018a). The deficiency of BMNCR has been reported to decrease bone mass, whereas the upregulation could stimulate bone formation and lower adipogenesis in the bone marrow. Moreover, BMNCR provided the scaffold to enhance the assembly of a transcriptional coactivator with a PDZ-binding motif (TAZ) and ABL to form TAZ and Runx2/peroxisome proliferator-activated receptor gamma (PPARG) transcriptional complexes, which are required for bone formation (Li et al., 2018a). Many enzymes protect the bone from oxidative stress, such as glutathione peroxidases (GPxs) and thioredoxin reductases (TrxRs), which play a crucial role in regulating bone homeostasis as well as reducing bone loss (Zhang et al., 2014). These enzymes contain selenocysteine, and selenocysteine insertion sequence (SECIS) binding protein 2 (SBP2) is an essential transacting factor required for the cotranslational insertion of selenocysteine into selenoproteins (Dumitrescu et al., 2010). It is reported that LncRNA Bmcob could control the transport of SBP2 between the nucleus and cytoplasm, and when overexpressed, Bmcob could regulate the expression of selenoproteins, sepp1, and increase osteogenesis of BMSCs (Sun et al., 2018). LncRNA-Neighboring enhancer of FOXA2 (NEF), which is known to the inhibit Wnt/β-catenin signaling and transforming growth factor-ßTGF-β pathways in cancer cells (Liang et al., 2018; Ju et al., 2019) is also reported in postmenopausal osteoporosis (PMOP) patients in a high concentration. In human BMSCs isolated from PMOP, enforced expression of NEF inhibited the secretion of IL-6, indicating its potential not only in diagnostics, but also as a prognostic biomarker for PMOP (Ma et al., 2019).

#### LncRNA Involved in BMSC Regulation Through miRNA

**FIGURE 2:**
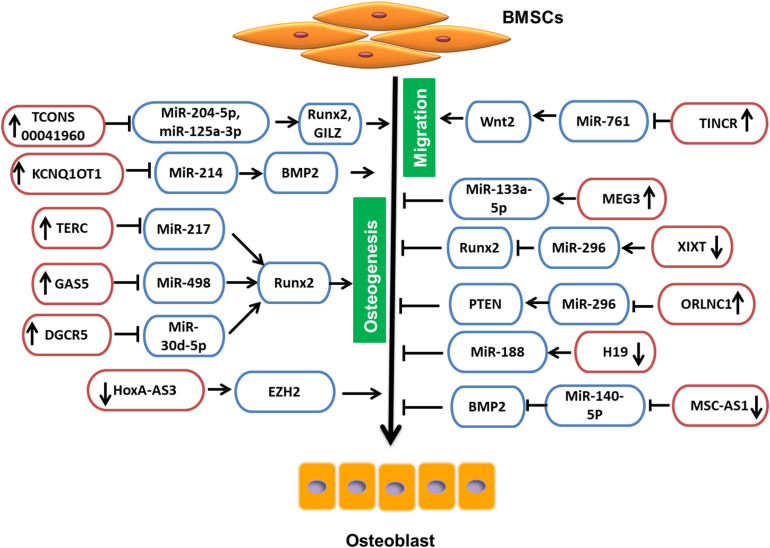
LncRNA involved in BMSC regulation by targeting miRNAs.

Due to their intrinsic ability of self-renewal and differentiation, BMSCs can be an ideal choice in treating osteoporosis. The available pharmaceutical interventions, though they have efficacy, also have side effects. Glucocorticoids are considered as standard therapy not only for reducing inflammation, but also for activation of the immune system in various diseases. Nevertheless, glucocorticoids have severe side effects on many organ systems, such as gastrointestinal, cardiovascular, endocrine, musculoskeletal, etc. (Oray et al., 2016). The most common cause of glucocorticoid administration is secondary osteoporosis and non-traumatic osteonecrosis (Weinstein, 2012). Glucocorticoid-treated BMSCs, when induced for differentiation, have shown low levels of TCONS 00041960 lncRNA. When overexpressed, TCONS 00041960 had a positive effect on the expression of osteogenic genes but not on the expression of adipocyte-specific genes. Moreover, TCONS 00041960 interactions with miR-204-5p and miR-125a-3p regulated not only Runx2, but also glucocorticoid-induced leucine zipper (GILZ), an antiadipogenic gene to control the differentiation fate of BMSCs treated with glucocorticoid (Shang et al., 2018). During osteogenic differentiation of BMSCs, lncRNA MSC antisense RNA 1 (MSC-AS1) and BMP2 were significantly upregulated. The 3′UTR of MSC-AS1 and BMP2 genes have been reported to act as a docking site for miR-140-5p. The inhibition of MSC-AS1 could decrease the expression of genes associated with osteogenesis as well as miR-140-5p, whose knockdown reduced BMP2 expression, but inhibition of both MSC-AS1 and miR-140-5p could rescue this inhibitory effect. Thus, miR-140-5p could not bind to BMP2, and MSC-AS1 could stimulate the osteogenic differentiation of BMSCs to ease osteoporosis progression (Zhang et al., 2019c). Similarly, LncRNA KCNQ1OT1 expression was also increased during osteogenic differentiation. It is shown that, when KCNQ1OT1 was upregulated, it targeted miR-214, which is known to bind to 3′UTR of BMP2. Therefore, by targeting miR-214, KCNQ1OT1 positively regulated the osteogenic differentiation of BMSCs by upregulating BMP2 expression (Wang et al., 2019a; Figure 2).

The telomere is essential for cell survival, and it is maintained by the enzyme telomerase that functions by adding guanine-rich repetitive sequences to maintain the length of telomeres (Zvereva et al., 2010). To maintain the activity of telomerase, the template sequence is offered by LncRNA TERC (telomerase RNA elements). According to Gao et al. (2020a), upregulation of lowered TERC could target miR-217, lower its levels, and indirectly increase Runx2, thus promoting osteogenesis and reducing the progression of osteoporosis. According to another study, an lncRNA, growth arrest-specific 5 (GAS5), was also present at a low concentration in osteoporosis patients and could also upregulate Runx2. When overexpressed, GAS5 increased hMSC osteogenic differentiation by targeting miR-498, which is known to target Runx2 and lower osteogenesis. Thus, by targeting miR-498, GAS5 could promote bone formation (Feng et al., 2019). Similarly, the study by Wu et al. reports the upregulation of lncRNA DiGeorge syndrome critical region gene 5 (DGCR5) in hMSCs derived from PMOP patients could promote hMSCs to differentiate to osteoblasts, increasing Runx2 expression though miR-30d-5p (Wu et al., 2018c).

LncRNA small nucleolar RNA host gene 1 (SNHG1) sponges the action of the number of miRNAs in cancer cells; similarly, lncRNA also plays an important role in the differentiation of osteoblasts and osteoclasts. The change in these miRNAs could lead to the loss of ability, which may indicate the possible involvement of SNHG1 in osteoporosis (Huang et al., 2019a). The overexpression of lncRNA SNHG1 is shown to reduce the differentiation ability of BMSCs by promoting the interaction between neural precursor cell expressed developmentally downregulated protein 4 (Nedd4) and p-p38 and p-p38 ubiquitination (Jiang et al., 2019b). Nedd4is an E3-ubiquitin ligase involved in the ubiquitination of membrane proteins and in regulating the availability of ion channels and transporter membrane proteins during signaling as well as enhancing bone formation (Jeon et al., 2018; Manning and Kumar, 2018). Thus, silencing of SNHG1 increased the bone mineral density (BMD) and levels of osterix (OSX) protein in OVX mice (Jiang et al., 2019b).

In the pathogenesis and development of PMOP, lncRNAs also play a significant role. The study reports that the enforced lncRNA maternally expressed gene 3 (MEG3) expression is positively correlated with the expression of miR-133a-3p in BMSCs derived from PMOP. This regulated expression of miR-133a-5p by MEG3 inhibited BMSC differentiation into osteoblasts (Wang et al., 2017a). Also, according to Zhuang et al. (2015), in MSCs isolated from multiple myeloma (MM), lncRNA MEG3 was present in a relatively low concentration during osteogenic differentiation. When MEG3 was knocked down, considerably lower expression of osteogenic markers as well as BMP4 were observed, whereas upregulation increased their expression (Zhuang et al., 2015). Another study on MSCs revealed that the lncRNA HOXA cluster antisense RNA 3 (HoxA-AS3) level was proportional to the adipogenic induction of MSC. However, the silencing of HoxA-AS3 in MSCs increased osteogenesis. Furthermore, the study revealed that HoxA-AS3 could interact with the enhancer of zeste 2 (EZH2) that is essential for Runx2 H3 lysine-27 trimethylation (H3K27me3). Thus, through epigenetic control, HoxA-AS3 could determine the fate of MSCs (Zhu et al., 2016). Metastasis-associated lung adenocarcinoma transcript 1 (MALAT1) is one of the lncRNAs that is associated with human diseases and is a major player in the development and pathogenesis of various cancers. In various mammalian species, MALAT1 is evolutionarily conserved and highly expressed (Zhang et al., 2017). LncRNA MALAT1 has also been reported in osteoporosis. MiR-143, which targeted the 3′-UTR of OSX, is reported to be the target of MALAT1. In addition, inhibition of MALAT1 is shown to downregulate the expression of OSX and reduces osteogenic differentiation, and overexpression could increase osteogenic differentiation (Gao et al., 2018), whereas, according to Zhang et al., inhibition of lncRNA MALAT1 is shown to reduce ALP activity in BMSCs and inhibit osteogenic differentiation of BMSCs via the MAPK signaling pathway (Zheng et al., 2019). Adipose-derived mesenchymal stem cells (ADSCs) act as a vital source of stem cells in tissue repair and regeneration and are shown to be regulated by MALAT1. The expression of MALAT1 has been reported to promote osteogenic differentiation of ADSCs by increasing Runx2 expression. Furthermore, a bifluorescein assay reveals that MALAT1 could target miR-30, which inhibits Runx2 (Yi et al., 2019).

For many medical conditions as well as fractures, glucocorticoids, such as dexamethasone (Dex), are commonly prescribed, but their long-term use leads to glucocorticoid-induced osteoporosis (GIOP; Whittier and Saag, 2016; Adami and Saag, 2019). One study has described that, when osteoblastic cell lines (OB-6 and hFOB1.19) were treated with Dex, the expression of lncRNA MALAT1 was downregulated. Furthermore, it showed that lentivirus-mediated upregulation of MALAT1 promoted cell viability and survival and inhibited PPM1E (protein phosphatase, Mg^2+^/Mn^2+^-dependent 1E), which activated AMPK signaling (Fan et al., 2018). Moreover, overexpression of MALAT1 also stimulated nicotinamide adenine dinucleotide phosphate (NADPH) activity and activation of Nrf2 signaling, whereas silencing intensified cytotoxicity induced by Dex. Thus, MALAT1 could shield the osteoblasts from Dex injury to promote cell survival (Fan et al., 2018). LncRNA epigenetically induced lncRNA 1 (EPIC1) has also been described by Zhang et al. (2018) to provide a protective effect on the survival of Dex-treated osteoblasts. The work has shown that the upregulation of EPIC1 stimulated cell survival and targeted Myc while silencing aggravated cytotoxicity induced by Dex (Zhang et al., 2018). Thus, through targeting different molecules and pathways, lncRNAs could regulate proliferation, differentiation, and functions of osteoblasts and even provide protection.

The osteoporosis-related lncRNA 1 (ORLNC1), which was highly expressed not only in BMSCs or the serum of osteoporotic mice, but also in bone tissue of osteoporotic patients, could decrease the osteogenic differentiation of BMSCs (Yang et al., 2019). Moreover, ORLNC1 is demonstrated to be a competitive endogenous RNA (ceRNA) for miR-296, which, by targeting phosphatase and tensin homolog (PTEN), a well-known negative regulator of osteogenesis could reduce osteogenesis (Yang et al., 2019).

Bone morphogenetic protein 1 (BMP1) is a metalloprotease that plays an important role in various cellular functions and acts as an extracellular matrix protein. It is vital in the process of osteogenesis, and its deficiency can increase bone fragility (Asharani et al., 2012). BMSCs isolated from osteoporosis patients show low expression of BMP1, which is regulated in miR-29b-3p. LncRNA nuclear enriched abundant transcript 1 (NEAT1) is shown to target miR-29b-3p and increase BMP1 in hBMSCs to promote osteogenic differentiation (Zhang et al., 2019e). Conversely, lncRNA XIXT is reported to reduce osteogenic differentiation of hBMSCs *in vitro* by reducing Runx2 through miR-30a-5p (Zhang et al., 2019b).

At the start of bone repair, an inflammatory response plays an important role in employing MSCs and directing not only their migration, but also differentiation, to speed up the process of bone repair (Liu et al., 2018). Therefore, it is important to understand the molecular mechanisms involved in regulating cell migration. Terminal differentiation-induced lncRNA (TINCR) has been reported in the proliferation and apoptosis as well as in cell migration (Zheng et al., 2020). Moreover, TINCR upregulation promoted cell migration of rat MSCs and also reduced miR-761, which targeted Wnt2. Thus, TINCR acted as a competitive endogenous RNA to increase the Wnt2 to promote migration of rat MSCs (Zheng et al., 2020). According to the study, MCF2L-AS1 was highly expressed during BMSC osteogenic differentiation, and knockdown could enhance the expression of miR-33a and reduce Runx2 expression. This could lead to inhibition of osteoblast differentiation. Thus, MCF2L-AS1 stimulated osteogenic differentiation in BMSCs by positively regulating the expression of Runx2 (Chen et al., 2020).

Long non-coding RNA, H19 was originally defined as an oncofetal transcript, expressed from the maternal allele (Ghafouri-Fard et al., 2020). In malignant tumors, it is well known that H19 is aberrantly expressed and regulates cell proliferation, migration, invasions, antiapoptosis, etc., as well as acts as a microRNA sponge to indirectly regulate the expression of microRNA target genes (Ye et al., 2019). Wang et al. (2018a) have described that the lncRNA H19 and Ligand-dependent co-repressor (LCoR) were upregulated in mBMSCs under osteogenic induction, and miR-188 was downregulated, which regulated LCoR in mBMSCs. Subsequent investigation has elucidated that, when H19 was silenced, the levels of miR-188 were increased, leading to reduced osteogenic differentiation in mBMSCs (Wang et al., 2018a). LncRNA H19 has also been reported to be influenced by mechanical tension. The mechanical strain could promote BMSCs for osteogenic differentiation as well as the expression of lncRNA H19 and lower the expression of miR-138. The deficiency of H19, however, reduced this mechanical tension-induced differentiation of BM-MSCs to osteoblasts and enhanced miR-138 expression, which weakened the focal adhesion kinase (FAK; Wu et al., 2018a). According to Huang et al. (2015), upregulation of H19 promoted *in vitro* osteogenic differentiation of hMSCs and bone formation *in vivo*. Furthermore, H19 along with miR-675 reduced the expression of transforming growth factor-β1 (TGF-β1), which inhibited Smad3 phosphorylation as well as histone deacetylase (HDAC) 4/5 to enhance expression of the osteoblast gene (Huang et al., 2015). Thus, by targeting different microRNAs, lncRNAs could regulate the different aspects of BMSCs and, consequently, affect osteogenesis (Table 2).

**TABLE 2:** LncRNA involved in BMSC regulation by targeting miRNAs.

LncRNA	Relative expression	Role	Target	References
TCONS 00041960	Upregulation	Promotes osteogenic differentiation of BMSCs	MiR-204-5p, miR-125a-3p	Shang et al., 2018
MSC-AS1	Downregulation	Inhibits osteogenic differentiation of BMSCs	MiR-140-5p	Zhang et al., 2019c
KCNQ1OT1	Upregulation	Stimulates osteogenic differentiation of BMSCs	MiR-214	Wang et al., 2019a
TERC	Upregulation	Increases osteogenesis of hMSCs	MiR-217	Gao et al., 2020a
GAS5	Upregulation	Promotes osteogenesis of hMSCs	MiR-498	Feng et al., 2019
DGCR5	Upregulation	Promotes osteogenesis of hMSCs	MiR-30d-5p	Wu et al., 2018c
SNHG1	Upregulation	Inhibits osteogenesis of BMSCs	Nedd4 and p-p38	Huang et al., 2019a; Jiang et al., 2019b
MEG3	Upregulation	Inhibits osteogenesis of BMSCs	MiR-133a-5p	Wang et al., 2017a
HoxA-AS3	Downregulation	Increases MSCs osteogenesis	EZH2	Zhu et al., 2016
MALAT1	Downregulation	Inhibits osteogenic differentiation of BMSCs	MiR-143, MPK Pathway	Gao et al., 2018; Zheng et al., 2019
MALAT1	Upregulation	Promotes cell viability and survival in OB-6 and hFOB1.19 cells	PPM1E	Fan et al., 2018
EPIC1	Upregulation	Stimulates cell survival of Dex-treated osteoblasts	Myc	Zhang et al., 2018
ORLNC1	Upregulation	Increases the adipogenic differentiation of BMSCs	MiR-296	Yang et al., 2019
NEAT1	Upregulation	Promotes osteogenic differentiation of hBMSCs	MiR-29b-3p	Zhang et al., 2019e
XIXT	Downregulation	Reduces osteogenic differentiation of hBMSCs	MiR-30a-5p	Zhang et al., 2019b
TINCR	Upregulation	Promotes migration of rat MSCs	MiR-761	Zheng et al., 2020
MCF2L-AS1	Upregulation	Stimulates osteogenic differentiation in BMSCs	MiR-33a	Chen et al., 2020
H19	Downregulation	Inhibits osteogenic differentiation	MiR-188	Wang et al., 2018a

#### LncRNA Involved in BMSC Regulation Through Wnt/β-Catenin Signaling

**FIGURE 3:**
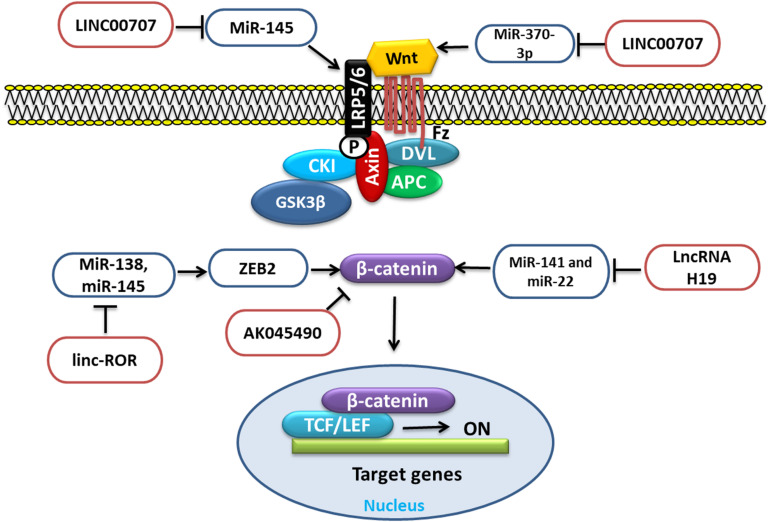
LncRNA involved in BMSC regulation through Wnt signaling by targeting different miRNAs.

Wnt/β-Catenin signaling is an important pathway that influences the function of different cells, which is governed and regulated by different factors, such as proteins, miRNAs, and lncRNAs. Various lncRNAs regulate the Wnt/β-Catenin signaling, such as LncRNA H19, LncRNA LINC00707, LncRNA linc-ROR, LncRNA AK045490, etc., through different mechanisms and targets (Figure 3).

Long non-coding RNA H19 upregulation is reported to function as a competing endogenous RNA for miR-141 and miR-22. These miRNAs negatively regulate the Wnt/β-catenin pathway and osteogenesis. Thus, due to the repression of these two miRNAs, β-catenin could be derepressed, which eventually would stimulate the Wnt/β-catenin pathway and osteogenesis (Liang et al., 2016). WNT2B, a member of the WNT family of proteins, which is an important player in various processes, such as cell proliferation and differentiation as well as osteogenic differentiation. It has been reported that, during osteogenic induction of hBMSCs, LncRNA LINC00707 overexpression promoted the osteogenic differentiation *in vitro* as well as *in vivo* by repressing miR-370-3p. This repression increased the WNT2B expression, which was the target of miR-370-3p (Jia et al., 2019). Low-density lipoprotein-related receptor 5 (LRP5) is an important receptor for Wnt signaling through which the signal is transduced, and the loss of LRP5 can increase the loss of bone mass (Williams, 2017). According to the study, the upregulation of LINC00707 could promote the Wnt/β-catenin pathway by targeting miR-145. This regulates the expression of LRP5 and then activates the Wnt/β-catenin pathway and increases the osteogenic differentiation of hBMSCs (Cai et al., 2020). Zinc finger E-box-binding homeobox 2 (ZEB2) is a member of the Zfh1 family. It regulates cell proliferation, invasion, and migration as well as apoptosis and also epithelial-to-mesenchymal transition (EMT) in tumors. Importantly, it also regulates β-Catenin (Qi et al., 2012). MiR-138 and miR-145 through ZEB2 could negatively regulate osteogenesis. LncRNA linc-ROR upregulated during hBMSC osteogenic differentiation is shown to target miR-138 and miR-145. This antagonizing action of linc-ROR activated ZEB2, a target shared by these two miRNAs, which subsequently activated the Wnt/β-catenin pathway and promoted osteogenesis (Feng et al., 2018a). Elevated lncRNA AK045490 is also reported to inhibit osteoblast differentiation of BMSCs, and its inhibition *in vivo* rescued bone formation in OVX osteoporosis mice. Furthermore, it is revealed that AK045490 targeted β-catenin and restricted its translocation into the nucleus, leading to suppression of Runx2, T-cell specific transcription factor (TCF1), and lymphoid enhancer binding factor (LEF1) expression and inhibition of osteoblast differentiation (Li et al., 2019a).

### LncRNAs in Osteoclast Regulation

Osteoclast cells are derived from the monocyte–macrophage lineage and are responsible for resorbing and remodeling bone. In addition, osteoclasts also play an essential role as immune cells. Therefore, the molecular mechanisms involved in regulating not only the formation and functions of osteoclasts, but also their interactions with other cells can help understand their role in diseases (Boyce, 2013; Kylmaoja et al., 2016; Table 3).

**TABLE 3:** LncRNAs involved in osteoclast regulation.

LncRNA	Relative expression	Role	Target	References
AK077216	Upregulation	Increases formation and functions of osteoclasts	NFATc1	Liu et al., 2019a
MIRG	Upregulation	Stimulates osteoclastogenesis	MiR-1897	Ling et al., 2019
CRNDE	Upregulation	Stimulates proliferation of osteoclasts	PI3K/Akt pathway	Li et al., 2018b
LINC00311	Upregulation	Promotes proliferation and the differentiation of osteoclasts	Delta-like 3 (DLL3)	Wang et al., 2018b
TUG1	Upregulation	Stimulates proliferation and reduces apoptosis in mice osteoclasts	PTEN	Han et al., 2019
CASC11	Upregulation	Stimulates proliferation of osteoclasts	TNF-α	Yu et al., 2019
Bmncr	Upregulation	Lowers differentiation of osteoclasts and bone resorption	–	Chen et al., 2019a

*“–” Unknown.*

According to Liu et al. (2019a), lncRNA AK077216 levels were associated with the regulation of different functions of osteoclasts. *In vivo* analysis in OVX mice has revealed the role of AK077216. It is demonstrated that the overexpression of AK077216 could increase the expression of the transcription factor, nuclear factor of activated T-cells 1 (NFATc1), whereas it could subdue NFAT-interacting protein 45 (NIP45) expression. This led to increased formation and functions of osteoclasts and, in turn, increased bone resorption (Liu et al., 2019a). Another lncRNA, lncRNA-MIRG, is reported to stimulate osteoclastogenesis when overexpressed. The study has further revealed that the MIRG functioned as a competitive endogenous RNA for miR-1897, which targeted NFATc1 and, thus, partially controlled the negative effect of miR-1897 on NFATc1 (Ling et al., 2019). LncRNA colorectal neoplasia differentially expressed (CRNDE) was also prominently expressed in osteoclasts of osteoporosis patients. The inhibition experiment has demonstrated the negative role of CRNDE on the cell cycle and cell proliferation and protein expressions of P53 although high expression of CRNDE stimulated the proliferation of osteoclasts through the PI3K/Akt signaling pathway (Li et al., 2018b). Another study has described the role of LncRNA LINC00311 in the Notch signaling pathway. Delta-like 3 (DLL3) is shown to be targeted by overexpressed LINC00311 and could lower the DLL3 expression. This downregulation could enhance cell proliferation by reducing apoptosis and differentiation by initiating the Notch signaling (Wang et al., 2018b). This promotes proliferation and, therefore, could increase the rate of bone resorption and enhance osteoporosis.

Long non-coding RNA taurine upregulated gene 1 (TUG1), which plays an effective role in the development of human cancers; ankylosing spondylitis, etc., is also shown to be involved in osteoporosis. Its upregulation in mice osteoclasts is reported to reduce the expression of PTEN and could stimulate proliferation and reduce apoptosis in mice osteoclasts (Han et al., 2019). Similarly, lncRNA cancer susceptibility 11 (CASC11), an oncogenic lncRNA that is identified in several types of cancer, such as gastric, colorectal, etc., has been found in abundance in osteoporotic patients. Further investigation has shown that, when lncRNA CASC11 was upregulated, it exercised a positive effect on the expression of TNF-α in osteoclast, which is known to activate osteoclasts and enhance bone resorption (Yu et al., 2019). Conversely, in the marrow and spleen of osteoporosis mice, Bmncr lncRNA was downregulated, but upregulation is shown to lower the expression of osteoclast-marker genes, such as Atp6v0d2, Acp5, Ctr, and Mmp9, and differentiation of osteoclasts induced by RANKL. Moreover, it also reduced the ability of bone resorption, thus reducing osteoporosis progression (Chen et al., 2019a).

### LncRNAs in Osteoblast Regulation

Osteoblasts are primarily involved in the formation of bone through different pathways, such as the PI3K/Akt cell signaling pathway, MAPK pathway, Wnt/β-catenin signaling, etc. The PI3K/Akt cell signaling pathway plays an important role in inhibiting osteoporosis by stimulating proliferation, differentiation of osteoblasts, and bone formation (Xi et al., 2015). In osteoblasts of PMOP rats, lower levels of phospho-PI3K (p-PI3K), p-Akt, and p-phosphoinositide-dependent kinase-1(PDK1) have been reported to be regulated by lncRNA AK023948 (AK0). It is also shown that the phosphorylation level of AKT was increased in the osteoblasts overexpressing the AK0 gene, and downregulation and knocking out decreased AKT phosphorylation levels in osteoblasts (Wang et al., 2020b). LncRNA AK125437, on the other hand, is reported to regulate the osteoblasts through the MAPK pathway to influence the bone mineral density in postmenopausal osteoporosis rats through p-p38 (Wang et al., 2020a). For osteoblast differentiation, lncRNA, antidifferentiation ncRNA (ANCR) is described as an indispensable factor. ANCR upregulation is shown to inhibit not only proliferation, but also osteogenesis of PMOP osteoblast cells *in vitro* and osteoid formation *in vivo*. This inhibition is credited to the lowered expression of Runx2 by the association of lncRNA-ANCR and enhancer of zeste homolog 2 (EZH2; Cai et al., 2019), whereas the elevated expression of lncRNA UCA1 in the plasma of osteoporosis patients inhibited osteoblast function. The inhibition of UCA1 showed increased proliferation and differentiation of preosteoblast MC3T3-E1 cell lines by stimulating bone morphogenetic protein-2 (BMP-2)/(Smad1/5/8) signaling pathway (Zhang et al., 2019d). Some lncRNAs are also expressed under the influence of microgravity. One such example is osteoblast differentiation-related lncRNA under simulated microgravity (ODSM). ODSM is reported to prevent the apoptosis of osteoblasts and enhance osteoblast mineralization *in vitro* (Wang et al., 2020c). Furthermore, it is described that the apoptosis in MC3T3-E1 could be reduced cells, and differentiation could be promoted in MC3T3-E1 cells subject to microgravity by the increasing level of lncRNA ODSM. In addition, the supplementation of LncRNA ODSM in mice under microgravity conditions reduced the apoptosis in bone tissue and increased osteoblast activity (Wang et al., 2020c).

According to Liu et al., during osteoblast differentiation, lncRNA TUG1 as well as marker genes, such as Axin 2, Frizzled-2, Runx2, and β-catenin, were upregulated. It is concluded that the silencing of lncRNA TUG1 using short hairpin TUG1 (shTUG1) could lower the OCN, OPN, and ALP activity and proliferation capability of osteoblast in addition to marker genes (Liu et al., 2019c). Similarly, lncRNA, differentiation antagonizing non-protein coding RNA (DANCR) could also regulate the proliferation and differentiation of preosteoblast cells through Wnt/β-catenin signaling (Jiang et al., 2019a). *In vitro* experiments revealed the inhibition of DANCR was able to stimulate the expression of differentiation-related genes, such as Runx2, collagen type I alpha 1, OSX and mineralization. Moreover, DANCR could also promote β-catenin expression, thus activating the Wnt/β-catenin pathway and promoting preosteoblast cells to proliferate and differentiate into osteoblasts (Jiang et al., 2019a). LncRNA MEG3 silencing is also reported to stimulate preosteoblast MC3T3-E1 cells to proliferate and differentiate into osteoblasts. The inhibition showed increased levels of Wnt and β-catenin proteins, suggesting increased Wnt/β-catenin signaling in osteoblasts (Li et al., 2019b). In bone formation, mineralization of the matrix is an important step, which is secreted by osteoblasts. In such cells, according to the study, lncRNA H19 was upregulated. Subsequent investigations showed H19 could promote osteoblast mineralization by targeting miR-185-5p, which inhibited IGF1 expression, an important factor involved in matrix mineralization (Wu et al., 2019; Table 4).

**TABLE 4:** LncRNAs involved in osteoblast regulation.

LncRNA	Relative expression	Role	Target	References
AK023948	Downregulation	Reduces proliferation, differentiation of osteoblast	AKT	Wang et al., 2020b
ANCR	Upregulation	Inhibits the proliferation and osteogenesis of PMOP osteoblast	RUNX2	Cai et al., 2019
UCA1	Downregulation	Increases proliferation and differentiation of osteoblast MC3T3-E1 cells	BMP-2/Smad1/5/8 pathway	Zhang et al., 2019d
ODSM	Upregulation	Prevents apoptosis of osteoblasts and enhance osteoblast mineralization	–	Wang et al., 2020c
TUG1	Upregulation	Increases osteoblast differentiation	–	Liu et al., 2019c
DANCR	Downregulation	Promotes preosteoblast proliferation and differentiation into osteoblasts.	β-catenin	Jiang et al., 2019a
MEG3	Downregulation	Stimulates preosteoblasts MC3T3-E1 cells to proliferate and differentiate into osteoblast	Wnt and β-catenin	Li et al., 2019b
H19	Upregulation	Promotes osteoblast mineralization	MiR-185-5p	Wu et al., 2019

*“–” Unknown.*

In summary, lncRNAs play a significant role in bone metabolism and osteoporosis. Through different pathways, lncRNAs influence different aspects of bone cells, such as proliferation, differentiation, and survival, indicating their potential in pharmaceutical therapeutics.

## CircRNA

Circular RNA is another group of endogenous ncRNAs that are circular, were originally considered to be by-products of abnormal splicing, and have a transcript length of hundreds to thousands of nucleotides. The common circRNAs that have been discovered to date originate from various segments of a gene, such as exons in the coding region of a gene, 5′- or 3′-untranslated regions (UTRs), introns, and regions between two genes, antisense RNAs, etc (Huang et al., 2019c; Lei et al., 2019). The lack of a 5′ or 3′ end in circRNAs confers them the stability and resistance to exonuclease digestion (Bose and Ain, 2018). CircRNAs are exclusively expressed in the cytoplasm and among diverse species and are highly conserved. CircRNAs have a great many functions in cellular activities. They act as an miRNA sponge and regulate transcription as well as protein and peptide coding (Zhai et al., 2018). They are also implicated in various diseases, such as cancer, bone diseases, etc. Because of their high stability and tissue specificity as well as disease-specific expression, circRNAs function as potential biomarkers for both diseases and prognosis (Zhai et al., 2018).

Circular RNAs are covalently closed-loop structures that are formed through backsplicing, a type of alternative splicing (Ebbesen et al., 2017). Pre-mRNA can generate a linear or circular RNA. If pre-mRNA goes through canonical splicing, it leads to the formation of exon-included linear RNA, and if it undergoes backsplicing, it produces circular RNA. Both canonical splicing signals as well as spliceosomal machinery are essentially backspliced exon circularization (Chen and Yang, 2015). CircRNA formation is generally classified into two mechanisms, namely direct backsplicing and lariat intermediate or exon skipping (Jeck and Sharpless, 2014). In backsplicing, intervening introns and two unspliced introns within a transcript pair are spliced. The donor of the 3′ intron is attacked by a branch point in the 5′ intron and the backsplice is completed by the 3′ donor through attacking the 5′ acceptor, forming a circular RNA, whereas, in exon skipping, it forms a lariat containing an exon that is spliced to remove the intronic sequence, forming a circular RNA (Jeck and Sharpless, 2014).

### Circular RNA Involved in BMSC Regulation

**FIGURE 4:**
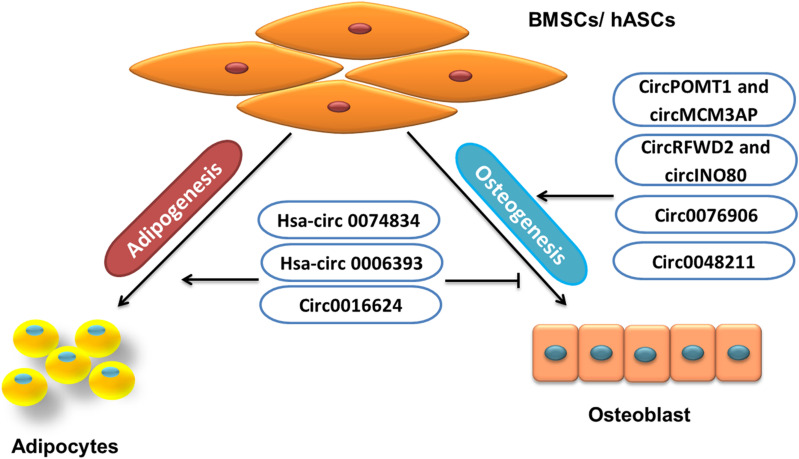
Role of circRNA in BMSCs/hASCs fate.

Some circRNAs inhibit osteogenic differentiation or promote adipogenesis of BMSCs/AMSCs, and others promote osteogenesis or inhibit the adipogenesis of BMSCs/AMSCs (Figure 4). Circular RNAs involved in BMSC regulation include hsa-circRNA 0074834, hsa-circRNA 0006393, circRNAs POMT1 and MCM3AP, circRNAs RFWD2 and INO80, circRNA 0076906, circRNA 0016624, circRNA 0048211, etc.

Bone fractures have the ability to repair themselves, but some bone fractures fail to heal, and in about 5% of cases, it leads to non-unions (Leighton et al., 2017). In such cases, the MSC-osteoblast lineage plays a significant role (Gibon et al., 2016). Some circRNAs help BMSCs toward osteogenesis, such as hsa-circ 0074834, which is reported to promote osteogenic differentiation of BMSCs and restore bone defects. The level of hsa-circRNA 0074834 in BMSCs from patients with bone fractures is reported to be decreased, and upregulation could target miR-942-5p to control the expression of E-Box binding homeobox 1 (ZEB1) and vascular endothelial growth factor (VEGF; Ouyang et al., 2019).

In BMSCs from GIOP patients, the downregulated expression of hsa-circRNA 0006393 has been reported. It is shown that hsa-circRNA 0006393 targeted miR-145-5p and also upregulated FOXO1 to increase the expression level of osteogenesis-associated genes (Wang et al., 2019b). Similarly, low expression of circRNAs POMT1 and MCM3AP is shown during hAMSc osteogenic differentiation. The loss-of-function experiment has revealed circRNA POMT1 and MCM3AP inhibition increased the expression of Runx2 and COL I. Furthermore, the study also showed increased expression of hsa-miR-6881-3p, which inhibited Smad6 and Chordin, the well-known inhibitors of BMP signaling. Thus, it can be concluded that the circRNAs POMT1 and MCM3AP could promote osteogenesis through BMPs signaling (Huang et al., 2019d). Another study has also reported the low levels of circRNA 0016624 during PMOP. The study resulted in establishing that the upregulation of circRNA 0016624 could induce BMP2 expression by acting as ceRNA to regulate the expression of miR-98, which previously has described targeting BMP2. Thus, circRNA 0016624 regulated the expression of miR-98 to increase osteogenic differentiation *in vitro* (Yu and Liu, 2019).

The bone formation and healing of bone defects can be promoted by a nel-like molecule, type 1 (NELL-1). The study conducted to investigate the effect of NELL-1 on the induction of osteogenesis in hASCs describes the high levels of circRNAs RFWD2 and circINO80 in hASCs. The inhibition of circRNAs RFWD2 and circINO80 showed reduced osteogenesis. Further investigation discovered that circRNAs RFWD2 and circINO80 interacted with hsa-miR-6817-5p, a negative regulator of osteogenesis. Thus, by sponging the action of has-miR-6817, circRNAs RFWD2 and circINO80 promoted osteogenesis (Huang et al., 2019b). Additionally, increased circRNA 0076906 expression during osteogenic differentiation of hMSCs is also shown to positively regulate osteogenesis. This positive regulation resulted from the sponging action of miR-1305, which targeted osteoglycin (OGN) to regulate osteogenic differentiation through the miR-1305/OGN pathway (Wen et al., 2020). Similarly, in another study, hBMSCs collected from PMOP patients undergoing osteogenic differentiation have shown upregulated circRNA 0048211, which also increased the expression of OCN, RUNX2, OPN, and ALP activity. Furthermore, the study has shown that circRNA 0048211 targeted miRNA-93-5p and consequently enhanced BMP2 expression (Qiao et al., 2020; Table 5).

**TABLE 5:** Role of circRNAs in BMSCs.

CircRNA	Relative expression	Role	Target	References
0074834	Upregulation	Inhibits osteogenic differentiation of BMSCs	MiR-942-5p	Ouyang et al., 2019
0006393	Upregulation	Inhibits osteogenesis	MiR-145-5p	Wang et al., 2019b
POMT1 and MCM3AP	Downregulation	Promotes osteogenesis of hASCs	Hsa-miR-6881-3p	Huang et al., 2019d
0016624	Upregulation	Inhibits osteogenesis	MiR-98	Yu and Liu, 2019
RFWD2 and INO80	Upregulation	Promotes osteogenesis of hASCs	Hsa-miR-6817-5p,	Huang et al., 2019c
0076906	Upregulation	Promotes osteogenic differentiation of hMSCs	MiR-1305	Wen et al., 2020
0048211	Upregulation	Increases osteogenic differentiation of hBMCs	MiR-93-5p	Qiao et al., 2020

### CircRNA Involved in Osteoclast Regulation

In bone marrow monocyte/macrophage (BMM) cells, the elevated expression of circRNA 28313 indicates when cells were induced. When circRNA 28313 was knocked down *in vitro*, differentiation of osteoclasts was repressed. In addition, it also inhibited bone resorption in OVX mice (Chen et al., 2019b). The study additionally employed the bioinformatics tool to further investigate the role of circRNA 20313. It demonstrated that circRNA 28313 and CSF1 acted as a binding site for miR-195a and collectively formed a circRNA–miRNA–mRNA network. Furthermore, it showed that circRNA 28313 functioned as ceRNA in targeting miR-195a to promote CSF1 and to regulate the osteoclast differentiation in BMM cells (Chen et al., 2019b).

### CircRNA Involved in Osteoblast Regulation

In osteoblasts and stromal cells, collagen type XV (ColXV), a bone extracellular matrix (ECM) protein helps to maintain potency and to promote differentiation (Lisignoli et al., 2017). The study has described that differentiation-induced MC3T3-E1 and MDPC23 osteoblasts exhibited increased mmu-circRNA 003795 and OPN expression. It further reports that when mmu-circ003795 was inhibited, expression levels of COL15A1 and OPN were decreased but that of miR-1249-5p was elevated. Thus, it indicates that, through miR-1249-5p, mmu-circRNA 003795 could target COL15A1 to regulate osteoblast differentiation and mineralization (Wu et al., 2020). For the activation of PI3K/Akt signaling and the phosphorylation of Akt, class I PI3K regulatory subunit 1 (α) (PIK3R1) is required (Cui et al., 2016), which is reported to be increased during fracture healing. The study suggests the PIK3R1 expression was proportional to the increased proliferation and lowered apoptosis of MC3T3-E1 cells. Moreover, in these cells, circRNA AFF4 silencing is shown to reduce Bcl-2 expression, an antiapoptotic protein, as well as PIK3R1. Furthermore, it showed circRNA AFF4 targeted miR-7223-5p, which inhibited PI3KR1, thus confirming circRNA AFF4 as a positive regulator of MC3T3-E1 proliferation (Mi et al., 2019). It is well known that reactive oxygen species (ROS) can kill bacteria as well as human cells. ROS also play an important role in diseases. It is not only the excessive amount that can lead to diseases, but a lack of ROS also contributes to the pathogenesis of diseases (Brieger et al., 2012). Reactive oxygen species, such as superoxide and hydrogen peroxide, significantly contribute to the progression of skeletal aging and various bone diseases (Callaway and Jiang, 2015). In hydrogen peroxide–treated human osteoblasts, reduced homeodomain-interacting protein kinase 3 (HIPK3) lncRNA expression was reported by Liang et al. (2020). The study demonstrated that the lentivirus-mediated circHIPK3 overexpression could increase cell viability in OB-6 osteoblastic and primary human osteoblast cells by inhibiting cell apoptosis. Furthermore, circHIPK3 silencing stimulated miR-124, which targeted cyclin-dependent kinase 6 and Rho-associated Protein Kinase 1 to increase cytotoxicity in OB-6 cells and primary human osteoblasts (Liang et al., 2020). In another study, the analysis of Dex-induced human osteoblasts and tissues of the necrotic femoral head of Dex-taking patients showed decreased cPWWP2A circRNA levels, but overexpression of cPWWP2A is shown to inhibit the miR-579, increasing the expression of SIRT1 and PDK1 (phosphoinositide-dependent protein kinase 1), the targets of miR-579 and cell death and apoptosis. Thus, by sponging, the actions of miR-579 cPWWP2A are shown to attenuate cytotoxicity in human osteoblasts (Hong et al., 2019; Table 6).

**TABLE 6:** Role and target of circRNAs in osteoblasts.

CircRNA	Relative expression	Role	Target	References
003795	Upregulation	Promotes osteoblast differentiation and mineralization	COL15A1	Wu et al., 2020
AFF4	Upregulation	Promotes MC3T3-E1 proliferation	MiR-7223-5p	Mi et al., 2019
HIPK3	Upregulation	Increases cell viability in OB-6 osteoblastic and primary human osteoblast cells	MiR-124	Liang et al., 2020
cPWWP2A	Upregulation	Inhibits apoptosis in osteoblast	MiR-579,	Hong et al., 2019

## Conclusion and Perspective

In this article, we have reviewed the different roles of lncRNAs and circRNAs in different bone cells and osteoporosis. The different lncRNAs and circRNAs in the serum of diseased patients as well as different bone cells can act as biomarkers. Many lncRNAs and circRNAs also act as competitive endogenous RNAs for many microRNAs to regulate the expression of microRNA targeted genes affecting different pathways that are important in the regulation of BMSCs, osteoblasts, osteoclasts, and osteogenesis. Some lncRNA and circRNAs that are upregulated in disease can be targeted to inhibit their expression although those that are downregulated can be stimulated to express sufficiently to increase bone formation.

Due to their abundance and ability to influence the different aspects of bone metabolism, lncRNAs and circRNAs have been shown as biomarkers in various diseases. Many reports mention lncRNAs, such as MALAT1 and SOX2-OT as potential biomarkers in various bone diseases, such as osteosarcoma (Wang et al., 2017b; Liu et al., 2019b) and lncRNA SNHG5, and CRNDE in acute myeloid leukemia (Li and Sun, 2018; Wang et al., 2018c), etc. Some reports mention exosomes that are rich in circRNA as a promising biomarker for diagnosing cancers (Li et al., 2015), such as lung adenocarcinoma (Liu et al., 2019d), circRNA 0004277 in acute myeloid leukemia (Li et al., 2017), circRNA NT5C2 in osteosarcoma (Nie et al., 2018), cardiovascular disease, etc. Because of the high-throughput techniques, such as Chip Scanning Analysis, it has become possible to detect the expression profile of different circRNA in various diseases, such as osteoarthritis and Kashin-Beck disease (Wang et al., 2020d).

New therapeutics could also be developed by targeting these ncRNAs. Some reports suggest the use of ASO or siRNAs, miRNAs, and circRNAs in different diseases, such as cardiovascular or cancers (Holdt et al., 2018; Lucas et al., 2018; Gao et al., 2020b). The major problem that hinders the use of ncRNAs, including lncRNAs and circRNAs, as potential therapeutics is their stability and delivery to the target cells, but advances in chemical modifications and strategies have proved that delivery systems can be improved to reduce unwanted side effects (Patil et al., 2019).

Still, the fact remains that there is a lack of detailed study on circRNAs in osteoporosis. Therefore, future research in this area could assuredly help understand bone metabolism and osteoporosis. Furthermore, undoubtedly much research is required to uncover the potential of lncRNAs and circRNAs in clinical settings in developing therapeutics to treat not only bone-related diseases, but also other life-threatening diseases.

## Author Contributions

SP and YG: writing – original draft preparation. SP, KD, XZ, YG, and AQ: writing – review and editing. AQ: funding acquisition.

## Conflict of Interest

The authors declare that the research was conducted in the absence of any commercial or financial relationships that could be construed as a potential conflict of interest.
